# GA signaling protein LsRGL1 interacts with the abscisic acid signaling-related gene *LsWRKY70* to affect the bolting of leaf lettuce

**DOI:** 10.1093/hr/uhad054

**Published:** 2023-04-19

**Authors:** Li Chen, Chaojie Liu, Jinghong Hao, Shuangxi Fan, Yingyan Han

**Affiliations:** Department of Plant Science and Technology, Beijing Key Laboratory of New Technology in Agricultural Application, National Demonstration Center for Experimental Plant Production Education, Beijing University of Agriculture, Beijing 102206, China; Department of Plant Science and Technology, Beijing Key Laboratory of New Technology in Agricultural Application, National Demonstration Center for Experimental Plant Production Education, Beijing University of Agriculture, Beijing 102206, China; Department of Plant Science and Technology, Beijing Key Laboratory of New Technology in Agricultural Application, National Demonstration Center for Experimental Plant Production Education, Beijing University of Agriculture, Beijing 102206, China; Department of Plant Science and Technology, Beijing Key Laboratory of New Technology in Agricultural Application, National Demonstration Center for Experimental Plant Production Education, Beijing University of Agriculture, Beijing 102206, China; Department of Plant Science and Technology, Beijing Key Laboratory of New Technology in Agricultural Application, National Demonstration Center for Experimental Plant Production Education, Beijing University of Agriculture, Beijing 102206, China

## Abstract

A variety of endogenous hormone signals, developmental cues, and environmental stressors can trigger and promote leaf lettuce bolting. One such factor is gibberellin (GA), which has been linked to bolting. However, the signaling pathways and the mechanisms that regulate the process have not been discussed in full detail. To clarify the potential role of GAs in leaf lettuce, significant enrichment of GA pathway genes was found by RNA-seq, among which the *LsRGL1* gene was considered significant. Upon overexpression of *LsRGL1*, a noticeable inhibition of leaf lettuce bolting was observed, whereas its knockdown by RNA interference led to an increase in bolting. *In situ* hybridization analysis indicated a significant accumulation of *LsRGL1* in the stem tip cells of overexpressing plants. Leaf lettuce plants stably expressing *LsRGL1* were examined concerning differentially expressed genes through RNA-seq analysis, and the data indicated enhanced enrichment of these genes in the ‘plant hormone signal transduction’ and ‘phenylpropanoid biosynthesis’ pathways. Additionally, significant changes in *LsWRKY70* gene expression were identified in COG (Clusters of Orthologous Groups) functional classification. The results of yeast one-hybrid, β-glucuronidase (GUS), and biolayer interferometry (BLI) experiments showed that LsRGL1 proteins directly bind to the *LsWRKY70* promoter. Silencing *LsWRKY70* by virus-induced gene silencing (VIGS) can delay bolting, regulate the expression of endogenous hormones, abscisic acid (ABA)-linked genes, and flowering genes, and improve the nutritional quality of leaf lettuce. These results strongly associate the positive regulation of bolting with *LsWRKY70* by identifying its vital functions in the GA-mediated signaling pathway. The data obtained in this research are invaluable for further experiments concerning the development and growth of leaf lettuce.

## Introduction

Leaf lettuce (*Lactuca sativa* L.), an annual vegetable crop in the Asteraceae family, is the most-eaten leafy vegetable globally and is highly valued due to its edible and medicinal properties [1]. Leaf lettuce is mainly cultivated in greenhouses or open fields, and during the process of cultivation is prone to high-temperature bolting (premature production of flowering stems) [2, 3]. High temperatures accelerate bolting and cause bitter leaves, reducing quality and production, thus resulting in limited leaf lettuce sales [4, 5]. Hence, to increase yields as well as sales, the flowering and bolting of leaf lettuce needs to be delayed while the cooking quality of the product needs to be maintained [6].

The flowering of plants is a process that involves various pathways concerning hormone signaling molecules and floral homeotic genes, whose combined effect causes a significant developmental change in the shoot apical meristem [7]. Many elements control the development of flowers, including transcription factors, gibberellin (GA), jasmonic acid (JA), abscisic acid (ABA), salicylic acid, cytokinin, auxin (IAA), and ethylene [8]. For instance, increased GA sensitivity of *Arabidopsis* overexpressing *ScNAC23* was detected in comparison with the wild type (WT), with significant acceleration of flowering and senescence due to the presence of exogenous GA [9]. The flowering period can be delayed as a result of the floral inductive signal, the *FLOWERING LOCUS T* (*FT*) being transported to the shoot apical meristem, and suppressing the expression of *SUPPRESSOR OF OVEREXPRESSION OF CO1* (*SOC1*) [10]. The interplay between *FT* and *SOC1* is a critical regulatory mechanism that controls the timing of flowering in plants. Therefore, several environmental variables, in conjunction with endogenous genetic elements, influence the bolting and flowering of plants [11].

Among the plant hormones the GAs, a large group of tetracyclic diterpenes, perform a vital function in the development and growth of plants [12]. One of the major bioactive GAs, GA_3_, contains a C3-hydroxyl group and is generated from a basic diterpenoid carboxylic acid skeleton [13, 14]. GAs are phytohormones that play prominent roles in controlling stem elongation and floral induction [15]. Constitutive GA response mutants or GA-insensitive dwarfs have been employed to investigate the genes involved in GA signal transduction. These mutants enable the identification and characterization of genes that are linked to the transduction of the GA signal [16]. *Arabidopsis* GA-insensitive mutants, which can be either dominant or semi-dominant, are characterized by their reduced sensitivity to GA. In wheat, such mutants exhibit decreased height, while in maize the short-internode *D8* and *D9* mutants, as well as mutants with the gain-of-function *gibberellin-insensitive* (*gai-1*) allele, also display GA-insensitive phenotypes [16].

The agriculturally important GA response gene family DELLA is highly conserved [17]. The adoption of semi-dwarf plant types, many of which were subsequently shown to contain mutations in either GA homeostasis or DELLA proteins, was partly responsible for the increased cereal crop yields in the Green Revolution of the 1960s [18]. The DELLA proteins in *Arabidopsis*, such as REPRESSOR of ga1-3 (RGA), RGA-LIKE 1 (RGL1), RGL2, RGL3, and GA INSENSITIVE (GAI), perform various functions and redundantly inhibit the plant responses mediated by GA [12]. A family of putative transcription regulators, the GRAS family, has a domain subfamily known as DELLA (VHIID), whose members are encoded by *RGA* and *GAI* in Chinese cabbage [19]. Double null mutations, such as *gai-t6 rga-24*, in the *ga1-3* background of *Arabidopsis* result in a ‘wild-type’ or GA ‘overdose’ phenotype, characterized by the increased promotion of GA-induced processes, including apical dominance, flowering time, leaf expansion, and stem elongation [16]. However, a semi-dwarf phenotype with semi-dominant gain-of-function mutation appears as a result of domain mutation of *GAI* and *RGA* [20]. RGL2 and RGL1 function as negative regulators, whereby the expression level of the former is temporarily elevated during the imbibition of the dormant seed, thus affecting germination, and the latter regulates various GA responses, such as the development of flowers and the elongation of stems [16, 21].

According to previous studies, some progress has been made in regulating stem extension and flower development by GA signaling, but there are still many problems. Plant growth and development can be co-regulated by a variety of hormones (e.g. GA, IAA, JA, and ethene) and environmental factors (e.g. light) [22, 23]. In *Arabidopsis*, ABA promotes DELLA gene translocation [24]. DELLA can bind to *JAZ1*, a jasmonate pathway repressor, and subsequently affect jasmonic accumulation [25]. JA is involved in the regulation of stamen growth and development by influencing the downstream elements of the DELLA protein, regulating its stability and activity [26]. Studies have also shown that GA signaling can eliminate the inhibition caused by DELLA proteins by upregulating some specific genes in the salicylic acid, ethylene synthesis, and JA pathways, thereby affecting leaf senescence [27]. The plant hormones GAs and brassinosteroids (BRs) have been found to interact with each other through a few key components. These components include the transcription factors of BRs and members of the DELLA protein family. By interacting with these components, GA signaling can modulate BR responses, and vice versa [28]. DELLA protein is involved in the signal transduction of GA, IAA, ethylene, ABA, and jasmonate and is essential in the development and growth of the plant.

Regardless of the observations above, it is still largely unknown how the DELLA protein regulates bolting and flowering. Further investigation of the process at the molecular level and signaling pathways is needed. In conclusion, this experiment was conducted to further understand the role of the *LsRGL1* gene in the GA pathway and the mechanism involved in regulating the development and growth of leaf lettuce. The RNA-seq analysis of leaf lettuce plants that exhibited a stable expression of *LsRGL1* depicted considerable enrichment of differentially expressed genes (DEGs) in the ‘phenylpropanoid biosynthesis’ and ‘plant hormone signal transduction’ pathways. Eight related transcription factors were identified in the COG (Clusters of Orthologous Groups) functional classification, among which the expression of the ABA signaling-associated *LsWRKY70* transcription factor varied significantly. The results of yeast single-hybrid (Y1H) experiments and β-glucuronidase (GUS) and biolayer interferometry (BLI) assays showed that LsRGL1 could interact with the promoter region of *LsWRKY70*, which confirmed that the *LsWRKY70* gene performs a vital function in leaf lettuce in terms of its development and growth.

## Results

### Promotion of the development and growth of leaf lettuce plants by exogenous GA_3_

After several field experiments, two cultivars (bolting-resistant cultivar S24 and bolting-sensitive cultivar S39) with a difference of 50 days in field high-temperature bolting time were selected [29]. Transcriptome analysis was conducted on the leaves of the two cultivars at the bolting stage. The results showed that the relative expression differences of GA signal transduction-related DELLA genes in the two cultivars were >3-fold, which suggested that GA-signal transduction-related factors may perform a crucial role in bolting and flowering of leaf lettuce, which is worthy of further study.

**Figure 1:**
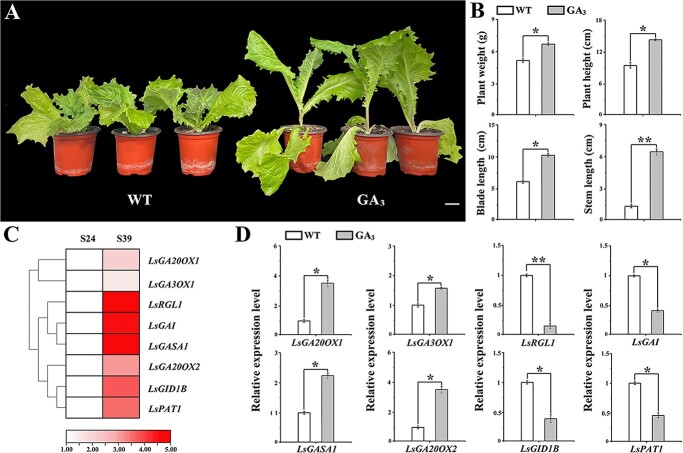
Identification of *LsRGL*1 as a candidate regulator of bolting in leaf lettuce. (**A**) Phenotype of S39 leaf lettuce after 150 mg L–1 GA_3_ treatment for 7 days. Scale bars = 5 cm. (**B**) Plant weight, plant height, leaf length, and stem length with WT and GA_3_ treatments. Scale bars = 5 cm. (**C**) Clustering heat map of GA transcription factors in the early stages of exogenous GA_3_ treatment. (**D**) After 7 days of GA_3_ application in S39, the expression levels of selected GA pathway genes were determined. The genes were *LsGA20OX2*, *LsGA3OX1*, *LsGASA1*, *LsGA20OX1*, *LsRGL1*, *LsGID1B*, *LsPAT1*, and *LsGAI*. Three biological replicates were performed, with 18S as the internal reference. Gene expression was determined by qRT–PCR utilizing three biological replicates. The standard error of the mean value (SD) is represented by error bars. ^*^P < .05, ^**^P < .01 (Student’s *t*-test).

To investigate the effect of GA on the growth and development of leaf lettuce plants, GA_3_ at a concentration of 150 mg L–1 was sprayed during the growth period of leaf lettuce. After 7 days of GA_3_ treatment, plant weight, plant height, blade length, and stem length of leaf lettuce plants were higher than those of WT (Fig. 1A and B). The difference in stem length was the most significant, indicating that exogenous GA_3_ increased bolting and accelerated the growth process of the leaf lettuce stem. After transcriptome analysis of S24 and S39 leaves during the bolting stage, eight genes with >3-fold relative expression differences were identified among 38 DEGs related to GA signal transduction (Supplementary Data Table S1). The association of the expression of the above-mentioned genes with the GA pathway was determined, the data indicating a strong link between the two (Fig. 1C). We designed primers and determined gene expression by qRT–PCR (Supplementary Data Table S2). The expression levels of *LsGA20OX2*, *LsGA3OX1*, *LsGASA1*, and *LsGA20OX1* increased after 7 days of GA_3_ treatment. However, *LsRGL1*, *LsGID1B*, *LsPAT*, and *LsGAI* decreased (Fig. 1D). The decrease in *LsRGL1* was the most significant. These data implied that exogenous GA_3_ hormone could enhance the development and growth of leaf lettuce (e.g. bolting) by increasing the expression of the genes linked to the process.

### *LsRGL1* regulates bolting of leaf lettuce

**Figure 2:**
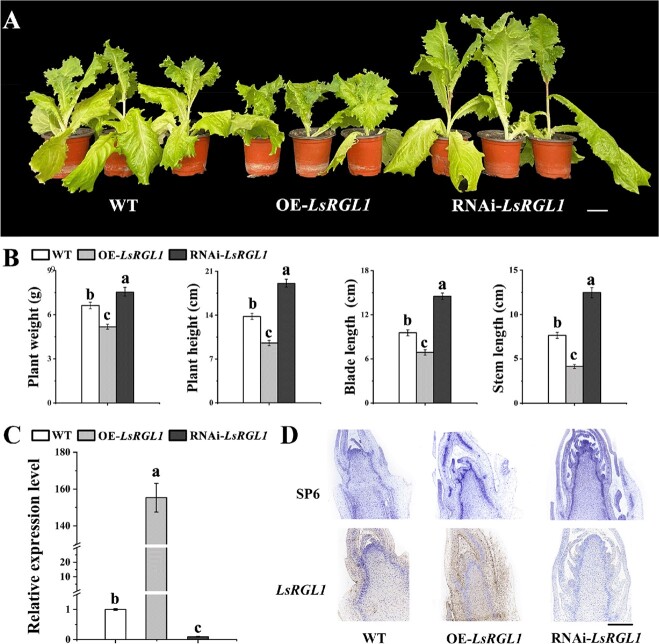
OE-*LsRGL1* inhibited leaf lettuce bolting, while RNAi-*LsRGL1* promoted bolting. (**A**) Expression of *LsRGL1* in WT, OE-*LsRGL1*, and RNAi-*LsRGL1*. Scale bars = 5 cm. ‘OE-*LsRGL1*’ represents the overexpression of the *LsRGL1* gene, and ‘RNAi-*LsRGL1*’ represents interference with the *LsRGL1* gene. (**B**) Plant weight, plant height, leaf length, and stem length of plants stably expressing *LsRGL1*. Scale bars = 5 cm. (**C**) The internal control was set as the 18S rRNA encoding gene. In stably expressing plants, the gene transcription level was examined by means of qRT–PCR to determine relative expression of *LsRGL1*. (**D**) *In situ* hybridization marker gene expression of WT, OE-*LsRGL1* and RNAi-*LsRGL1*. SP6 was the control respectively. Scale bars =100 μm. The three replicates’ standard errors of the mean are indicated by error bars. One-way analysis of variance (ANOVA) followed by Tukey’s multiple range test was used to determine the values with significant variation (*P* < .05), which are indicated by different letters placed above the bars.

To explore the function of the *LsRGL1* gene, *Agrobacterium tumefaciens* containing gene overexpression (OE) or interference vectors, OE-*LsRGL1* and RNAi-*LsRGL1*, respectively, was transformed into leaf lettuce plants. The data obtained indicated that plant weight and height, leaf length, and stem length of stably transformed OE-*LsRGL1* plants were considerably less than those of WT or RNA interference plants, whereas RNAi-*LsRGL1* plants were considerably larger than WT or overexpression plants. The plant height and stem length of RNAi-*LsRGL1* plants were twice those of OE-*LsRGL1* plants (Fig. 2A and B). The qRT–PCR results showed a considerably enhanced expression level of OE-*LsRGL1*, whereas RNAi-*LsRGL1* was considerably reduced in comparison with the control (Fig. 2C). *LsRGL1* was detected in the nucleus by subcellular localization (Supplementary Data Fig. S1). Additionally, *in situ* hybridization analysis showed that *LsRGL1* was expressed at stem tips, and the OE-*LsRGL1* signal was significantly enriched compared with RNAi-*LsRGL1* and WT plants. In contrast, RNAi-*LsRGL1* flower bud differentiation was more apparent (Fig. 2D). Thus, *LsRGL1* was identified as a negative regulator of bolting.

### Transcriptome analysis of leaf lettuce under stable expression and identification of genes with differential expression

To explore the molecular mechanism of *LsRGL1* stable transformation, extraction of total RNA from four biological replicates of OE-*LsRGL1*, RNAi-*LsRGL1*, and WT leaf lettuce was performed, and the data were utilized to generate cDNA libraries. We obtained 85.2 Gb of clean data (each sample at least 6.71 GB) by Illumina high-throughput RNA sequencing from 12 samples. A total of 31 447 unigenes were collected from public protein databases [KEGG (Kyoto Encyclopedia of Genes and Genomes), GO (Gene Ontology) and COG] and 27 838 unigenes were annotated. The total length of clean reads ranged from 45 058 344 to 50 573 638 among the different libraries, and the percentage of 30 bases was >94.65% after filtering out low-quality and rRNA reads (Supplementary Data Table S3).

**Figure 3:**
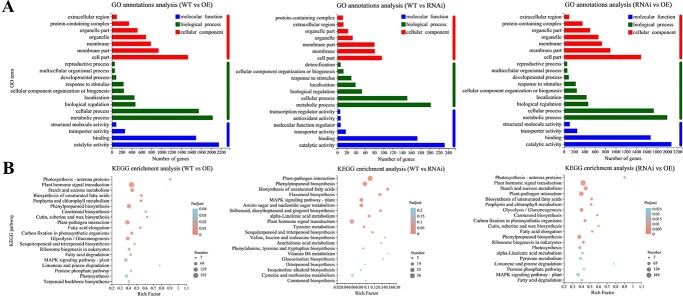
GO classification and KEGG pathway enrichment of DEGs in stable expression of lettuce leaves. (**A**) GO classification of DEGs. (**B**) KEGG pathway enrichment of DEGs. ‘OE’ represents OE-*LsRGL1*, and ‘RNAi’ represents RNAi-*LsRGL1*.

**Figure 4:**
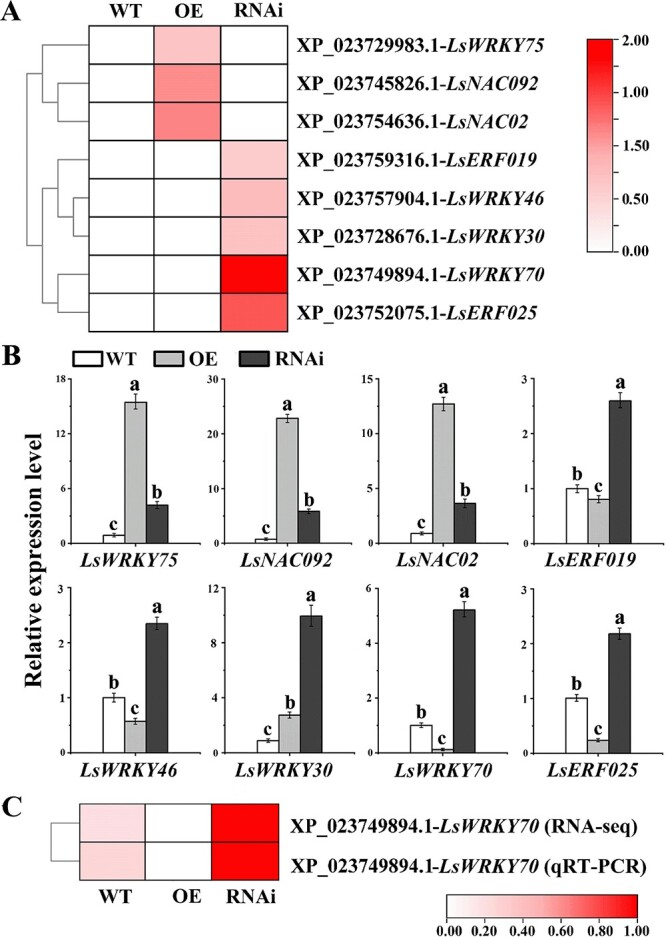
Identification and analysis of growth-linked genes. (**A**) Candidate growth-linked genes’ expression profiles are shown by heat maps. (**B**) RNA-seq expression profile-related validation by qRT–PCR. These parameters include *LsWRKY75*, *LsNAC092*, *LsNAC02*, *LsERF019*, *LsWRKY46*, *LsWRKY30*, *LsWRKY70*, and *LsERF025*. (**C**) RNA-seq and qRT–PCR data showing the correlation of *LsWRKY70* expression. ‘OE’ represents OE-*LsRGL1*, and ‘RNAi’ represents RNAi-*LsRGL1*. The three replicates’ standard errors of the mean are indicated by error bars. One-way analysis of variance (ANOVA) followed by Tukey’s multiple range test was used to determine values with significant variation (*P* < .05), which are indicated by different letters placed above the bars.

In the Venn diagram, there were 843 DEGs in WT, OE, and RNAi (Supplementary Data Fig. S2), where 152 genes were consistent with the expression profiles of RNAi-*LsRGL1* < WT < OE-*LsRGL1*, whereas 248 genes were consistent with the expression profiles of RNAi-*LsRGL1* > WT > OE-*LsRGL1*, subject to further categorization according to their GO annotations into various functional categories. The numbers of DEGs involved in ‘catalytic activity’ (CA), ‘molecular function’ (MF), ‘biological processes’ (BPs), and ‘metabolic processes’ (MPs) were the largest in the three groups under study (Fig. 3A). An increased number of the DEGs in most of the treatments were from the BP and MF categories. Notably, in the three groups, enrichment of KEGG terms linked to ‘phenylpropanoid biosynthesis’ and ‘plant hormone signal transduction’ was indicated (Fig. 3B). The DEGs were filtered according to an expression level |log2(fold-change)| > 1 and false discovery rate (FDR) < .05 in each pairwise comparison. We selected eight genes related to bolting and flowering out of 54 transcription factors with significant differences. At the same time, eight plant hormone signal transduction and response proteins, such as genes linked to ABA, NAC transcription factors, and ethylene-responsive genes, were annotated in COG (Fig. 4). The results of the study imply that *LsRGL1* may play a crucial role in the bolting process of leaf lettuce by regulating the expression of hormone-related genes.

### COG gene expression analysis

To verify the expression of hormone-related genes annotated from COG, the correlation analysis showed that the expression of these eight genes was closely correlated with bolting, with *LsWRKY70* (XP_023749894.1) having the strongest correlation (Fig. 4A). Eight representative genes were analyzed by qRT–PCR during leaf development of stably transformed OE-*LsRGL1* and RNAi-*LsRGL1*. The results showed upregulation of *LsWRKY75* (XP_023729983.1), *LsNAC092* (XP_023745826.1), and *LsNAC02* (XP_023754636.1) in OE-*LsRGL1* plants, but depicted the opposite trend in RNAi-*LsRGL1* plants. Expression levels of *LsERF019* (XP_023759316.1), *LsWRKY46* (XP_023757904.1), *LsWRKY30* (XP_023728676.1), *LsWRKY70*, and *LsERF025* (XP_023752075.1) were considerably reduced in OE-*LsRGL1* plants, but were significantly promoted in RNAi-*LsRGL1* plants (Fig. 4B). The transcriptome analyses of qRT–PCR and RNA-seq results were shown to exhibit strong correlation and integrity by analyzing the relative gene expression of the selected *LsWRKY70* (Fig. 4C). Therefore, the *LsRGL1* gene may affect lettuce bolting by regulating the related genes.

### Both *in vivo* and *in vitro* biochemical tests verified that LsRGL1 could bind *proLsWRKY70*

**Figure 5:**
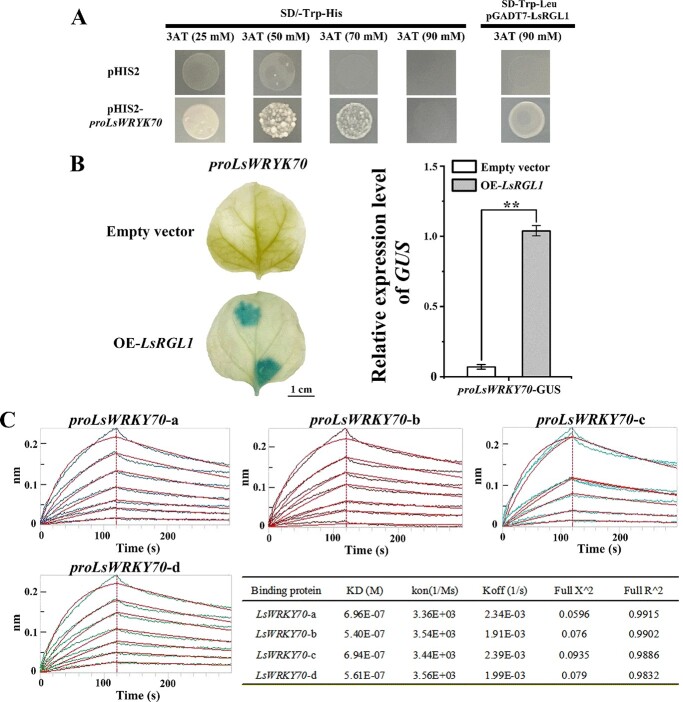
LsRGL1 proteins bind to *proLsWRKY70*. (**A**) Direct binding of LsRGL1 to *LsWRKY70* promoters demonstrated by Y1H assays. Yeast cells cotransformed with pGADT7 + pHIS2-*proLsWRKY70* were utilized as controls. The optimum 3-AT concentrations for inhibiting background histidine biosynthesis of the pHIS2 vector are shown by the values in parentheses. (**B**) Using the GUS reporter gene, a transient transactivation assay was performed on *N. benthamiana* leaves. Only after cotransformation with the LsRGL1 and LsWRKY70 promoters was GUS staining observed. Scale bars = 1 cm. GUS expression was detected with a GUS gene quantitative detection kit. GUS staining was carried out using three biological replicates. The standard errors of the mean values of the measurements of the three replicates are indicated by error bars. *P < .05, **P < .01 (Student’s *t*-test). (**C**) Binding affinities of LsRGL1 to the *LsWRKY70* promoters were quantified by BLI assay. The concentrations of the protein were 25 and 180 nM for the upper and lower lines, respectively. *proLsWRKY70*-a, *proLsWRKY70*-b, *proLsWRKY70*-c, and *proLsWRKY70*-d all bound to LsRGL1 protein, and *proLsWRKY70*-a, *proLsWRKY70*-b, and *proLsWRKY70*-d were concentration-gradient-dependent. However, the promoter *proLsWRKY70*-c showed no significant concentration gradient dependence.

To explore the molecular mechanism of LsRGL1 regulating leaf lettuce bolting-related genes, a Y1H assay was utilized to examine whether LsRGL1 binds to *proLsWRKY70*. LsRGL1 and *proLsWRKY70* were ligated into pGADT7 and the pHIS2 vector, respectively, for self-activation and subsequent detection. The results indicated an absence of HIS3 nutritional reporter gene expression in control or even in cells that contained other constructs, whereas yeast cells that simultaneously contained both the aforementioned constructs (pGADT7-LsRGL1 and pHIS2-*proLsWRKY70*) expressed the reporter gene (Fig. 5A). Therefore, we deduced that this gene represents likely LsRGL1 targets. *Nicotiana benthamiana* leaves were utilized for the transient transactivation assay, which was the next step in the investigation into the transcriptional control of *LsWRKY70* by LsRGL1. The coding sequence of LsRGL1 was inserted into a construct that contained the *35S* promoter of the cauliflower mosaic virus. This construct was cotransformed into *N. benthamiana* leaves alongside another construct that contained GUS protein fused with *LsWRKY70* promoters. An increase in the relative expression levels and staining of GUS was detected during the coexpression of LsRGL1 with *proLsWRKY70* (Fig. 5B). Finally, the potential binding was examined further through BLI. Using the GCC-boxes of *proLsWRKY70*, probes labeled with biotin were designed and were subsequently divided into a, b, c, and d segments. The results indicate the binding of each to LsRGL1 (Fig. 5C). The resulting kinetic values obtained from the BLI assay, which was utilized to measure the binding affinities of LsRGL1 to *proLsWRKY70*, suggested considerably strong interactions. Hence, it was confirmed that *LsWRKY70* is a direct target of LsRGL1.

The transcription level of genes is regulated by *cis*-acting elements in the upstream promoter sequence, while the induction conditions of *proLsRGL1* and *proLsWRKY70* are still unclear. To elucidate the transcriptional regulation mechanism of *proLsRGL1* and *proLsWRKY70*, the transcriptional regulation mechanism sequence for *cis*-acting elements was determined using PlantCARE (http://bioinformatics.psb.ugent.be/webtools/plantcare/html/). The resulting data showed that *proLsRGL1* included GA corresponding elements and elements responsive to light, ABA, auxin-, and methyl jasmonate (MeJA), as well as other factors (Supplementary Data Table S4). *proLsWRKY70* included elements that responded to light, ABA, MeJA and various other factors (Supplementary Data Table S5). This indicates that the expression of *proLsRGL1* and *proLsWRKY70* may be induced by the external environment (light) and phytohormones (GA, ABA, auxin, MeJA). The expression levels of *LsRGL1* and *LsWRKY70* were analyzed after treatment with light, GA, ABA, IAA, and MeJA, to establish a theoretical basis for revealing the role of the external environment and phytohormones in the regulation of lettuce biosynthesis. qRT–PCR results showed that, after treatment with the above factors, the expressions of *LsRGL1* and *LsWRKY70* were induced to different degrees (Supplementary Data Fig. S3). Studies have speculated that light, GA, ABA, IAA, and MeJA play important regulatory roles in lettuce biosynthesis.

### *LsWRKY70* silencing by virus-induced gene silencing delayed bolting and improved lettuce nutritional quality

**Figure 6:**
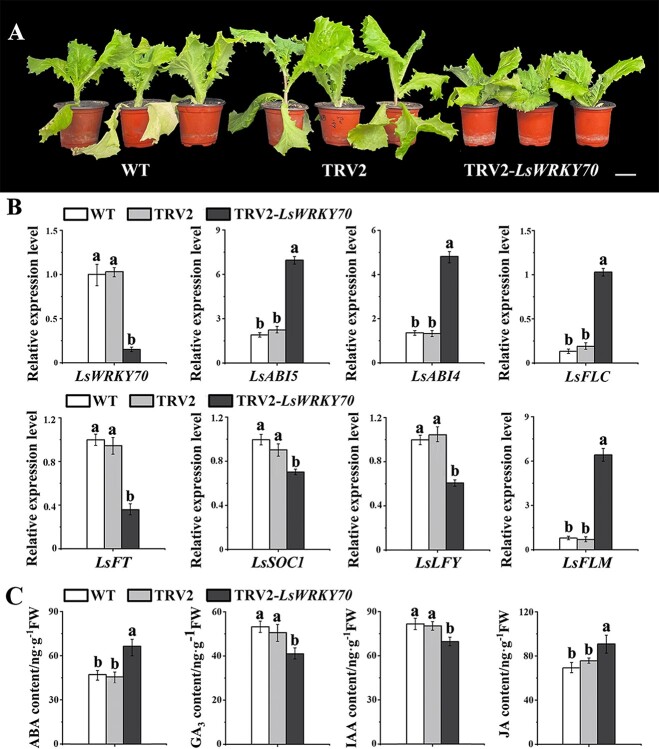
The TRV2-*LsWRKY70* gene in S39. (**A**) The bolting phenotype was developed 7 days after TRV2-*LsWRKY70* infection. Scale bars = 5 cm. (**B**) qRT–PCR was used to assess relative transcription levels of eight genes (*LsWRKY70*, *LsABI5*, *LsABI4*, *LsFU*, *LsLFT*, *LsSOC1*, *LsLFY*, and *LsFLM*) in leaf lettuce plants that had been inoculated with TRV2-*LsWRKY70*. (**C**) Contents of ABA, GA_3_, JA, and IAA. The standard errors of the mean from three replicate measurements are indicated by error bars. One-way analysis of variance (ANOVA) followed by Tukey’s multiple range test was used to determine values with significant variation (*P* < .05), indicated by different letters placed above the bars.

The role of the *LsWRKY70* genes was examined further by suppressing their expression in S39 using VIGS in the TRV vector. The research analyzed below confirms that *LsWRKY70* silencing can delay bolting while improving the nutritional quality of lettuce. Seven days after infection, leaf lettuce plants infected with the virus-carrying TRV2-*LsWRKY*70 showed a delayed bolting phenotype (Fig. 6A). qRT–PCR was employed for comparison and analysis of the gene transcription levels between TRV2-*LsWRKY70* plants and control plants. Regarding the expression levels of *LsWRKY70*, the data of three independent analyses depicted a significant reduction in the silenced plants, whereas during examination of genes of the ABA signal transduction pathway the results showed that the transcription levels of *ABSCISIC ACID*-*INSENSITIVE 4* (*ABI4*) and *ABSCISIC ACID*-*INSENSITIVE MUTANT 5* (*ABI5*) were considerably higher than those in the control plants. At the same time, flowering-related genes were detected, and the expression levels of *FT*, *SOC1*, and *LEAFY* (*LFY*) were decreased, while the expression levels of *FLOWERING LOCUS C* (*FLC*), and *FLOWERING LOCUS M* (*FLM*) were significantly upregulated compared with the control (Fig. 6B). Moreover, the levels of phytohormones were measured, showing that ABA and JA levels increased while GA_3_ and IAA levels decreased (Fig. 6C). The results showed that the delay in bolting following silencing could affect the content of phytohormones. Therefore, the expression of phytohormones and ABA-related genes in TRV2-*LsWRKY70* plants is important for lettuce bolting.

To verify the effect of TRV2-*LsWRKY70* on the nutritional quality of leaf lettuce, physiological data were analyzed. The leaf lettuce plants expressing silenced TRV2-*LsWRKY70* were significantly shorter than control plants, and plant weight, plant height, leaf length, and stem length were significantly smaller than WT or TRV2. When *LsWRKY70* was silenced, the contents of soluble sugar, malondialdehyde (MDA) and vitamin C (VC) of leaf lettuce were considerably reduced compared with the control. However, the cellulose content of leaf lettuce increased. The antioxidant enzyme activity assay conducted in leaf lettuce TRV2-*LsWRKY70* and control showed that phenylalanine ammonia lyase (PAL), catalase (CAT), and superoxide dismutase (SOD) activities increased steadily in both. There was no major variation between WT and TRV2, while a rapid reduction in peroxidase (POD) activity was detected (Supplementary Data Fig. S4). These results imply that TRV2-*LsWRKY70* could delay bolting and improve the nutritional quality of lettuce by regulating hormones and ABA-related genes.

## Discussion

### *LsRGL1* mediates gibberellin signaling during leaf lettuce bolting

Acting as a vital phytohormone, GA controls the regulation of various plant development processes [30]. The inhibitor of GA biosynthesis, uniconazole, causes yield increase in cauliflower and purple kale by suppressing bolting and flowering, whereas these two processes are promoted in Chinese cabbage, mustard, and radish through the functions of GA [15, 19, 31]. Researchers have documented that the flowering and bolting of leaf lettuce are influenced through DELLA proteins by GA, although the process has not been elucidated on the molecular level yet. In this study, GA_3_ at a concentration of 150 mg L–1 was sprayed during the growing period of leaf lettuce to induce rapid bolting, and the resulting data were congruent with prior research (Fig. 1). Monitoring their expression and correlation, the link between DELLA genes, flower-related genes, and cell elongation was investigated. Studies have shown that the expression of the DELLA genes is downregulated after a 3-hour GA treatment at the three-leaf stage of Chinese cabbage, indicating that these genes may inhibit stem growth, and degradation of this gene with GA_3_ can initiate stem elongation and development [19]. All *RGL1*-transgenic plants in *Arabidopsis* showed delayed bolting, consistent with a lack of GA biosynthesis or GA perception [16]. Studies have shown that the overexpression of *RGL1* or functional acquisition of *GAI* can partially save the early flowering phenotype of *WRKY75*-overexpressing plants in *Arabidopsis* [21]. During stamen development in pumpkin flowers, the transcription levels of GA receptor *GID1b* and DELLA inhibitor *GAIPb* increased significantly in mature flowers [32]. Therefore, we speculated that *LsRGL1* (a member of the DELLA family) inhibits stem growth and development, while GA_3_ treatment degrades it and initiates stem elongation and development. These results support the view that DELLA proteins act as inhibitors of GA-responsive plant growth (Fig. 2A). *LsRGL1* negatively regulates the GA response and plays an important role in controlling stem elongation [12, 16].

### *WRKY70* mediated regulation and crosstalk of hormones

The study of WRKY transcription factors and hormone crosstalk has recently advanced substantially. Research has shown that three groups of WRKY genes, *OsWRKY24*, *OsWRKY53*, and *OsWRKY70*, have the features of typical WRKY transcription factors, and negatively regulate the transcription of ABA and GA signaling [21, 33, 34]. Moreover, studies have shown that the *wrky75* mutation leads to delayed flowering in *Arabidopsis* and significantly accelerates flowering after overexpression, positively regulating flowering in an FT-dependent manner [21]. *SlWRKY23* is hypersensitive to ethylene, JA, and auxin-mediated root growth; the flowering time of the transgenic plants was shortened, and the plants showed more inflorescence branching [35]. In *Arabidopsis*, prior research has documented the link between DELLA and all three groups of WRKY proteins to examine the influence of GA on floral initiation [7, 21]. The exogenous application of GA_3_ influenced the flowering time, whereby the *wrky12* and *wrky13* mutants all showed different effects. GA_3_ sped up flowering time in the WT, whereas in comparison with the WT the phenotypic analysis depicted delayed flowering in the *wrky12* mutant and early flowering in the *wrky13* mutant, implying that the effect of the *wrky12* and *wrky13* mutations could be regarded as a positive and negative factors, respectively, in flowering-time regulation. The flowering modulated by GA shows partial dependence on the *wrky* mutations [36]. Moreover, this study found that in *LsRGL1* transgenic plants, KEGG analysis detected enrichment of genes related to ‘phenylpropanoid biosynthesis’ and ‘plant hormone signal transduction’ (Fig. 3B). The data of these analyses indicated the vital function of hormones concerning their impact on bolting. Additionally, it was hypothesized that *LsWRKY70* could promote ABA synthesis and activate ABA-dependent signaling pathways.

A recent study showed that the bZIP transcription factor genes *ABI5* and *ABI4* play a negative role in ABA-mediated *LsFLC* transcription inhibition and are also key components of ABA signal transduction pathways [37]. The relative expression levels of *LsABI4*, *LsABI5*, and *LsFLC* showed that silencing *LsWRKY70* enhanced the tolerance of transgenic lines to osmotic stress. Hence, we speculated that this was due to the interaction of *LsWRKY70* with flowering-related genes and ABA signaling pathways (Fig. 6B). In *Arabidopsis*, *ABI5* was found during the screening of ABA-insensitive mutants, and ABA has a strong induction effect on *ABI5* nutritional expression [38]. In addition, *FLC* was shown to be the target of *ABI4* and *ABI5*; *FLC* expression was directly regulated by these two genes, thus precisely controlling flower transformation in plants [39, 40]. In Chinese cabbage, *SOC1* overexpression accelerated early flowering and stem elongation, while *SOC1* knockout significantly delayed bolting and flowering [40]. These findings imply that the tolerance of the transgenic lines may be improved via the ABA signaling pathway, thereby delaying bolting, and that these effects can be linked to the elevation of the expression levels of these genes by *LsWRKY70.* The data obtained in this study were consistent with previous studies, where it was reported that overexpression of *OsWRKY72* disrupted signal cross-talk between ABA signals and auxin transport pathways [40]. Additionally, early flowering and decreased apical dominance were demonstrated in *OsWRKY72* transgenic *Arabidopsis*.

### Effect of hormone crosstalk on bolting of leaf lettuce

Hormones play a vital function in all aspects of the development and growth of plants. In lettuce, for instance, stems exposed to the combined effect of GA and auxin increased in thickness in comparison with those treated by GA solely at the same stage of flower bud differentiation. This increased thickness was associated with the presence of IAA, which promoted the elevation of GA_3_ content, leading to further stimulation of stem elongation and growth [41]. Several hormonal cues, including cytokinin and GA, control the start and progression of the stalk-forming process in Chinese cabbage [30]. This study found that GA affected lettuce bolting and flowering by influencing ABA. The data suggested that exogenous hormone administration can control the breeding cycle and offer more effective breeding methods. Therefore, it is essential to further elucidate how exogenous hormones can regulate the mechanism of lettuce bolting.

### *WRKY70* silencing treatment affects physiological changes in leaf lettuce

Through long-term co-evolution and ongoing natural selection, plants have gradually modified their survival strategies to cope with harsh climatic circumstances. This adaptation includes alterations in numerous physiological and metabolic pathways, such as the removal of reactive oxygen species (ROS), an increase in the synthesis of protein-protective complex, a decrease in osmotic regulators, variation in carbohydrate metabolism, and increased energy and lipid metabolism [42–44]. Moreover, the accumulation of soluble proteins, soluble sugars, and vitamin C can promote osmotic potential and play an important metabolic role in stress tolerance [44–46]. Soluble proteins help to safeguard enzyme function, stabilize protein synthesis, and control cytoplasmic acidity and alkalinity [47]. Soluble sugars, as binding substances of membrane lipids, play a critical role in maintaining membrane stability, and vitamin C is a major contributor to antioxidant capacity [47, 48]. The findings of this study are congruent with the findings of earlier publications (Supplementary Fig. S4B). Following *LsWRKY70* silencing treatment, soluble protein and sugar, as well as vitamin C content, increased to improve the stability and protect the integrity of the cell membrane of leaf lettuce by increasing the content of osmoregulatory substances.

Oxidative stress is caused by environmental stress, which interferes with cells’ normal metabolism, causing ROS to accumulate through an imbalance of the formation and clearance of ROS [49]. The antioxidant enzymes SOD, CAT, and POD are produced under stress situations to scavenge harmful ROS, thus resulting in increased tolerance to these stressors [41, 50, 51]. The data of this research is congruent with prior studies (Supplementary Fig. S4C). These results indicate that an extra response mechanism to stress was established by *LsWRKY70* silencing-related treatment during leaf lettuce bolting and the enzymatic system was activated, thereby further enhancing the stress-adaptive ability, to improve the quality of leaf lettuce.

This research has identified the molecular pathways via which *LsWRKY70* controls bolting. These findings show that *LsWRKY70* interacts with LsRGL1 protein through ABA hormone-mediated bolting in leaf lettuce as a novel member of the bolting regulatory network. Further research into the regulation of *LsWRKY70* transcription and translation during the switch from vegetative to reproductive growth will be intriguing. The conservation of this mechanism for other bolting-associated WRKY transcription factors is also a prospective area of research. This research has indicated the regulation of the onset and progression of bolting by *LsWRKY70* as a novel member of the ABA-mediated signaling pathway.

## Materials and methods

### Plant materials and growing conditions

The bolting-sensitive *L. sativa* cultivar S39, provided by Cathay Green Seeds (Beijing) Co., Ltd, was planted in the greenhouse of the Beijing University of Agriculture Experimental Station. Approximately 200 disease-free seeds were selected and sown in 50-hole disks. The growth conditions of lettuce were as published previously [5]. When the seedlings had grown to four leaves and one heart they were transferred to a 9-cm pot.

### Exogenous GA_3_ treatment

Following the ripening of the lettuce seedlings, a concentration of 150 mg L–1 GA_3_ solution once every 3 days was sprayed onto them for a total of two times.

### Plant transformation

Lettuce S39 plants were stably transformed by *Agrobacterium*-mediated transformation using vectors containing overexpression and interference genes, OE-*LsRGL1* and RNAi-*LsRGL1*. First, plump seeds were selected, sterilized, and grown on the medium for 3 days. The resulting leaves were cut with a blade, placed in a medium, and grown for 2 days in dark conditions. Next, the leaves were soaked in *Agrobacterium* solution for 15 minutes, followed by 2 days of growth in dark conditions. The extremely important process of transferring the leaves t o the medium for screening for resistance until new shoots emerged was carried out carefully. Finally, the shoots were placed in the rooting medium and transplanted when the roots were strong. The same parts of leaves from lettuce were extracted and wrapped in tinfoil. Primers from those listed in Supplementary Data Table S2 were used to screen the transformed plants.

### *In situ* hybridization

Fresh specimens were fixed with RNA-free FAA fixative for 24 hours, routinely dewaxed, and washed. Anti-peeling slides were utilized, and the cover slides of cell crawling slides were treated with polylysine, fixed for 20–30 minutes, fully washed with diethy l pyrocarbonate (DEPC) water, dried naturally, and frozen at −20°C for >2 weeks. The wet box comprised 5 × SSC (pH 7.5) (35 mL) + formamide (35 ml). Instructions for the chromogenic in situ hybridization (CISH) *in situ* hybridization kit were referred to for specific steps.

### RNA-seq library preparation and sequencing and transcriptome assembly and gene annotation

RNA libraries were prepared and sequenced, and 12 sequenced libraries were generated using the Illumina HiSeq platform (Illumina, Inc., CA, USA). Assembly and gene annotation were conducted according to previously published methods [5, 52]. The sequences of *LsGA20OX1* (TR7985|c0_g1_i2), *LsGA3OX1* (TR26290|c0_g1_i1), *LsRGL1* (TR1754|c0_g1_i1), *LsGAI* (TR1809|c0_g1_i1), *LsGASA1* (TR35751|c0_g1_i1), *LsGA20OX2* (TR40399|c0_g1_i3), *LsGID1B* (TR26207|c0_g1_i1), and *LsPAT1* (TR28459|c0_g1_i1) were aligned with the homologous sequences in the CoGe database (https://genomevolution.org/CoGe/).

### Yeast one-hybrid assay

The *LsWRKY70* promoter sequence was cloned into the pHIS2 vector at polyclonal sites (EcoRI and BamH1) in front of the HIS3 reporter gene. The corresponding LsRGL1 transcription factors were cloned on the pGADT7 vectors (EcoRI and BamH1). A specific concentration of 3-amino-1,2,4-triazole (3-AT) was added to inhibit the background expression of this reporter gene, and suppressed the growth of false-positive bacteria. The Y1H technique was conducted as reported previously [53] (Supplementary Data Tables S1 and S2).

### β-Glucuronidase staining assays for transient expression in leaf lettuce

The *LsWRKY70* promoter fragment (1793 bp) was cloned into the pBI101 vector (BamHI and SmaI sites). At the same time, the LsRGL1 transcription factor sequence was cloned into the pBI121 vector (XbaI and SacI sites). The transient expression of these genes in lettuce was conducted using the *Agrobacterium*-mediated method, and GUS staining then determined their expression using prior research methods (Huayang Biology, China) [54].

### Biolayer interferometry assay

BLI assays were conducted using an octet RED 96 system (Forte Bio, USA) with His-Tag sensors. The LsRGL1-His and GST-pGADT7 solutions were diluted to 20 μg ml^−1^ at a pH value of 4.5 using 10 mM sodium acetate, and the dilutions were employed to soak the sensors for 10 minutes. The dilution of the purified *proLsWRKY70* samples in BLI buffer to different concentrations was carried out to serve as analytes [54], according to the directions of the manufacturer. BLI was used to determine the dissociation constants (*K*_D_) and the on-and-off rate for HIS-LsRGL1 binding to GST-*LsWRKY70*.

### Construction of and infection with *LsWRKY70* virus-induced gene silencing vectors

The LsWRKY70 fragment was inserted into the TRV2 vector, creating a 291-bp insertion fragment. To design the primers for this insertion, specific DNA sequences recognized by the BmaH1 and EcoR1 restriction enzymes were incorporated as limiting sites (Supplementary Data Table S2). Following the successful connection of the insertion fragment with the TRV2 vector, the resulting construct was transferred to *Agrobacterium* strain GV3101 to facilitate its introduction into the cells. The experiment was divided into a blank control group (WT), a negative control group (TRV2), and an experimental group (TRV2-*LsWRKY70*) according to previously published methods [5].

### Malondialdehyde content and soluble sugar concentration

Soluble sugar content The soluble sugar test kit (Thermo Fisher Technology Co., Ltd., China) was utilized to perform the test per the instructions of the manufacturer. Then, 2 mL 10% (w/v) trichloroacetic acid (TCA) was added to 0.5 g of fresh lettuce lea ves, ground into a uniform mixture, and centrifuged. Afterward, 0.6% (w/v) thiobarbituric acid (TB A) was added to the supernatant liquid (volume 2ml), followed by 15 min in a 95°C water bath, pla ced on top of the ice, and centrifuged. The absorbance values of the supernatant at 450 nm were det ermined. The quantitative formula is as follows: soluble sugar concentration (mmol L^–1^)=11.71 × A_450_.

### Vitamin C (VC) content

The VC assay kit (Nanjing Jiancheng Institute of Biological Engineering, China) was utilized for testing according to the directions of the manufacturer. R1 (0.45 ml) was added to 0.15 g fresh lettuce leaves. The mixture was ground evenly and centrifuged. The supernatant was separated, followed by the addition of R2, R3, and R4. The mixture was vortically mixed and subjected to incubation at 37°C for 30 minutes. Finally, the supernatant was isolated. Absorbance at 536 nm was measured, and the quantitative formula used for VC content (μg ml^−1^) was (measured OD value − blank OD value)/(standard OD value − blank OD value) × 6 × 4.

### Cellulose content

The determination of cellulose content was performed using the Cellulose Content Detection Kit (Solarbio, China), following the manufacturer’s instructions. To be precise, 0.1 g of lettuce leaves was utilized, and the test was carried out according to the protocol. Subsequently, the spectrophotometer was calibrated to a wavelength of 620 nm, and distilled water was used to adjust it to 0 nm.

### Soluble protein content

The experiment was conducted according to the instructions of a soluble protein detection kit (Thermo Fisher Technology Co., Ltd, China). Initially, a standard curve was drawn with protein content as the abscissa and absorbance as the ordinate. Then, 5 ml buffer was added to 0.5 g fresh lettuce leaves. The mixture was ground to a uniform consistency and centrifuged. Next, 0.3 ml of supernatant was introduced into the test tube, followed by the addition of 5 ml of Coomassie Brilliant Blue reagent and appropriate shaking. Following a 2-minute rest period, the color at 595 nm was compared, the absorbance was calculated, and the protein content was checked using the standard curve. Finally, the following equation was used to calculate protein content: soluble protein content = C × VT/VS × WF × 1000 (mg/g).

### Enzyme-linked immunoassay

In this experiment, an enzyme-linked immunoassay (ELISA) kit (Thermo Fisher Technology Co., Ltd, China) was used for detection and analysis [6]. Lettuce leaves (0.1 g) were ground into a powder with liquid nitrogen following the directions of the kit. The absorbance of GA_3_, ABA, IAA, and JA at 492, 490, 490, and 450 nm was determined, with the optical densities increased by 0.01 unit.

## Acknowledgements

We appreciate Cathay Green Seeds (Beijing) Co., Ltd for supplying testing materials. For his wise counsel, Dr Ji Tian (Beijing University of Agriculture, China) is gratefully acknowledged. The Beijing Joint Research Program for Germplasm Innovation and New Variety Breeding (G20220628003), The Beijing Natural Science Foundation Program (22JF0014), and The National Natural Science Foundation of China (3177110780) all contributed financially to the present project.

## Author contributions

L.C.: software, verification, methodology, and writing original draft. C.L.: resources, software, supervision, and project management. J.H.: methodology and validation. S.F.: supervision, resources, and seeking funding. Y.H.: conceptualization, writing review and editing, and project administration.

## Data availability

The datasets generated for this study can be found in the NCBI SRA database: PRJNA880996 and SRP076512.

## Conflict of interest

The authors declare that they have no conflict of interest.

## Supplementary data

Supplementary data is available at *Horticulture Research* online.

## Supplementary Material

Web_Material_uhad054
