# Supplementary material for: GA signaling protein LsRGL1 interacts with the abscisic acid signaling-related gene LsWRKY70 to affect the bolting of leaf lettuce
Source: Hortic Res. 2023 Apr 19;10(5):uhad054. doi: 10.1093/hr/uhad054 (PMC10199715; doi:10.1093/hr/uhad054)
Supplement: Web_Material_uhad054 [file web_material_uhad054.zip › Supplementary Fig.docx]

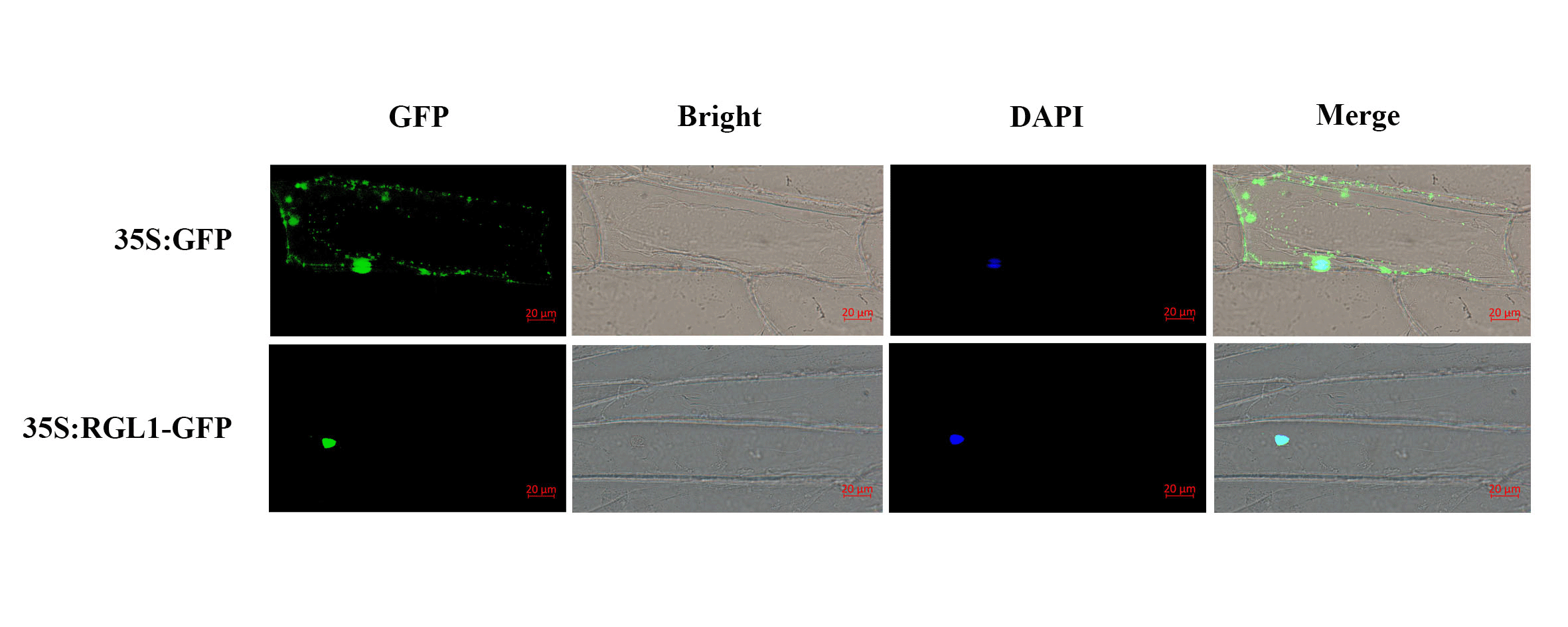


**Supplementary Fig. S1.** Subcellular localization of LsRGL1 fusion protein in Allium cepa cells. Plasmid with green fluorescent protein (GFP) alone served as the control. Bar = 50 μm.


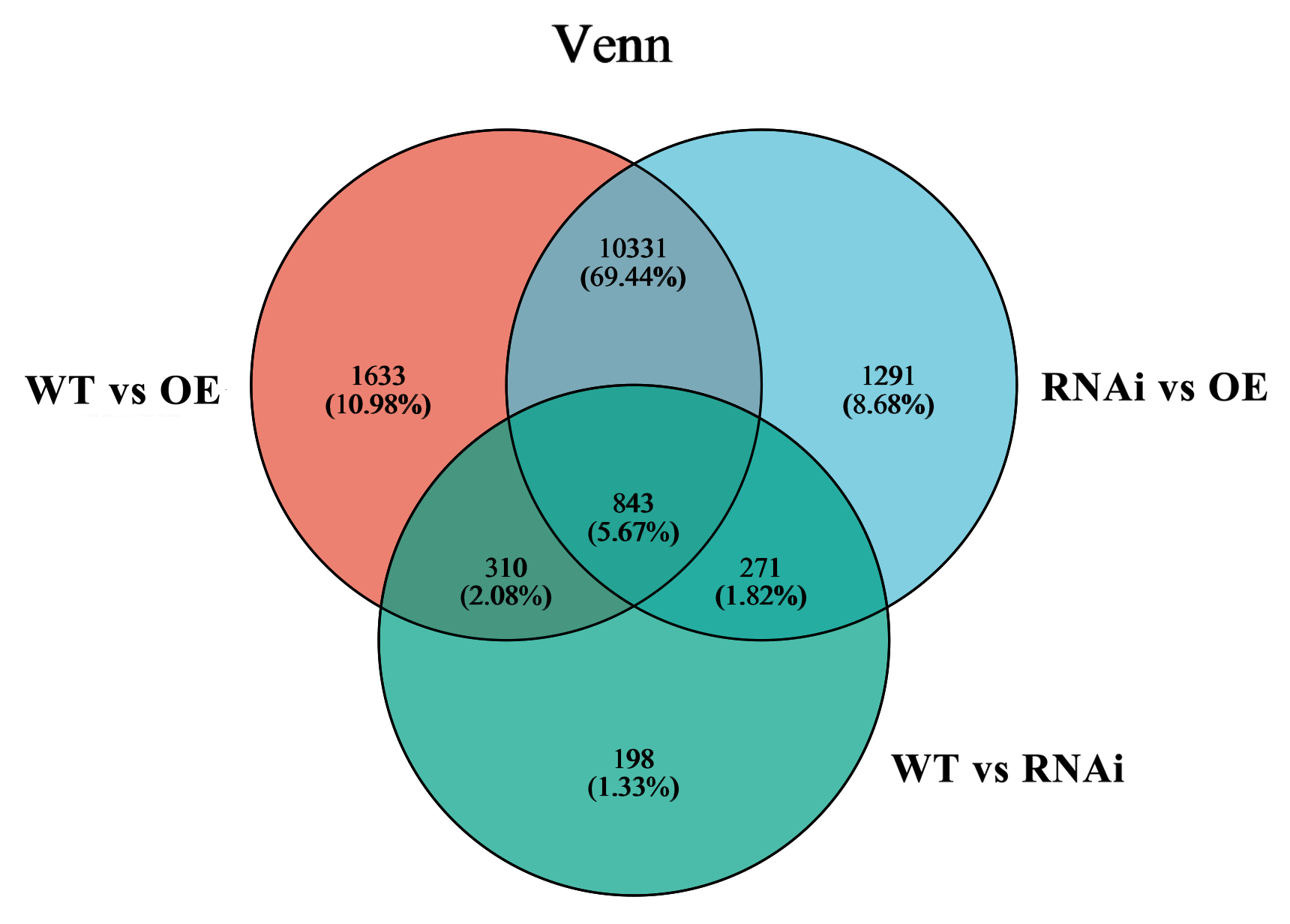


**Supplementary Fig. S2.** Venn diagram of the RNA-seq data WT, OE and RNAi.


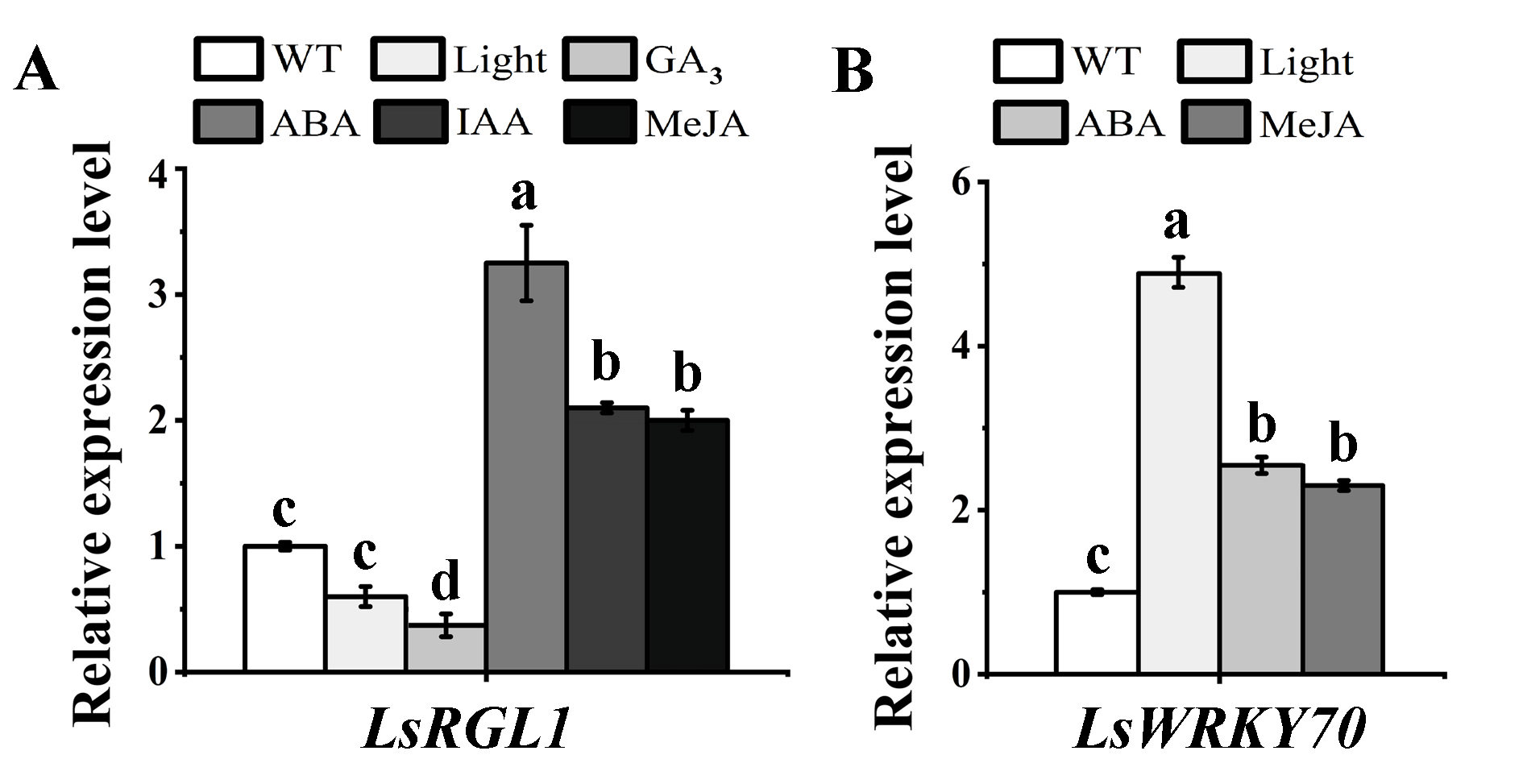


**Supplementary Fig. S3.** Expression levels of *LsRGL1* and *LsWRKY70* in lettuce treated with Light, GA, ABA, IAA, and MeJA. The expression levels of *LsRGL1* after treatment with light, GA, ABA, IAA, and MeJA were detected by qRT-PCR. The expression levels of *LsWRKY70* after treatment with light, ABA and MeJA were determined by qRT-PCR. The three replicates’ SEM is indicated through error bars. Different letters above the bars indicate significantly different values (*P* < 0.05) calculated using one-way analysis of variance (ANOVA) followed by Tukey’s multiple range test.


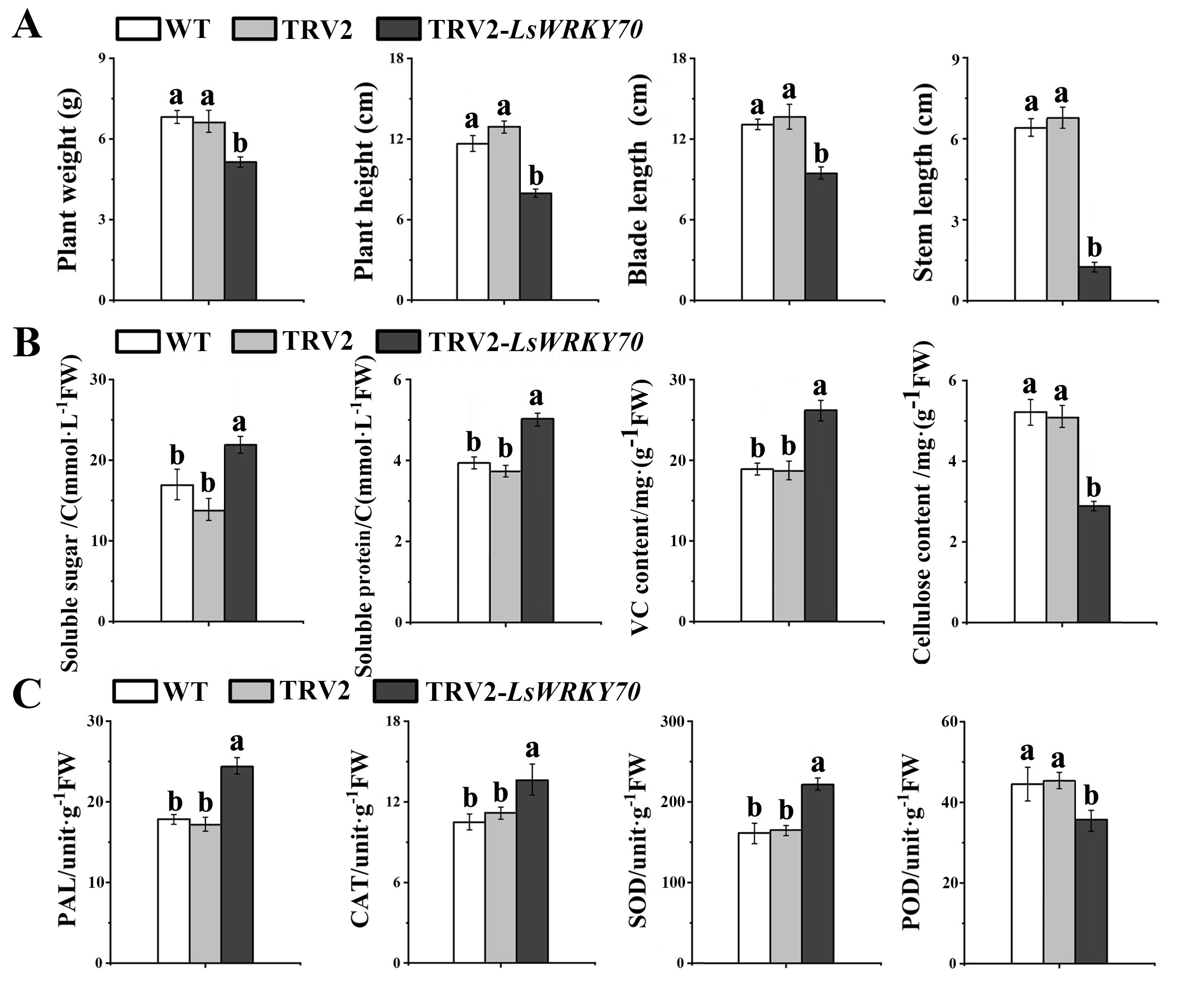


**Supplementary** **Fig. S4.** The TRV2-*LsRGL1* gene affects the nutritional quality of leaf lettuce. (A) The plant weight, plant height, leaf length, and stem length of leaf lettuce with TRV2-*LsWRKY70* treatments. Scale bars = 5 cm. (B) Changes in soluble sugar content, MDA content, VC content, and cellulose content in lettuce leaves after TRV2-*LsWRKY70* treatment. (C) Activities of PAL, CAT, SOD, and POD during TRV2-*LsWRKY70* treatment leaf lettuce. A 0.01 increase in absorbance at each value of 290 nm, 240 nm, 560 nm, and 290 nm per min was denoted as one unit (U) of PAL, CAT, SOD, and POD activity. The SEM of three replicate measurements is indicated through error bars. One-way analysis of variance (ANOVA) followed by Tukey's multiple range test, was used to determine the values with significant variation (*P* < 0.05), which were indicated through different letters placed above the bars.
