# Supplementary material for: GA signaling protein LsRGL1 interacts with the abscisic acid signaling-related gene LsWRKY70 to affect the bolting of leaf lettuce
Source: Hortic Res. 2023 Apr 19;10(5):uhad054. doi: 10.1093/hr/uhad054 (PMC10199715; doi:10.1093/hr/uhad054)
Supplement: Web_Material_uhad054 [file web_material_uhad054.zip › Supplementary Table.docx]

**Supplementary Table S1 The number of access to eight gene sequences**

| **GeneID** | **Rice-gene-id** | **Ath-gene-id** | **Gene-name** | **Accession numbers** |
| --- | --- | --- | --- | --- |
| TR7985\|c0_g1_i2 | LOC_Os03g63970.1 | AT4G25420.1 | GA20OX1 | Lsat_1_v5_gn_4_37400.1 |
| TR26290\|c0_g1_i1 | LOC_Os01g08220.1 | AT1G15550.1 | GA3OX1 | Lsat_1_v5_gn_5_133201.1 |
| TR1754\|c0_g1_i1 | LOC_Os03g49990.1 | AT1G66350.1 | RGL1 | Lsat_1_v5_gn_2_69481.1 |
| TR1809\|c0_g1_i1 | LOC_Os03g49990.1 | AT1G14920.1 | GAI | Lsat_1_v5_gn_4_13640.1 |
| TR35751\|c0_g1_i1 | LOC_Os05g35690.1 | AT1G75750.1 | GASA1 | Lsat_1_v5_gn_4_74180.1 |
| TR40399\|c0_g1_i3 | LOC_Os04g39980.1 | AT1G14130.1 | GA20OX2 | Lsat_1_v5_gn_2_72821.1 |
| TR26207\|c0_g1_i1 | LOC_Os05g33730.1 | AT3G63010.1 | GID1B | Lsat_1_v5_gn_2_65001.1 |
| TR28459\|c0_g1_i1 | LOC_Os10g22430.1 | AT5G48150.1 | PAT1 | Lsat_1_v5_gn_5_122860.1 |

**Supplementary Table S2 DNA primers used in this study**

| **Primer name** | **Primer Sequence (5’-3’)** | **Used for** |
| --- | --- | --- |
| OE-LsRGL1-F | ATGAAGAGAGAGCACAAC | Gene cloning |
| OE-LsRGL1-R | TTCCACACGATTGATTCG | Gene cloning |
| RNAi-LsRGL1-F | CGCACTTACAGGCGATTA | Gene cloning |
| RNAi-LsRGL1-R | GGTGTGAGCGTCGCAGAA | Gene cloning |
| proLsRGL1-F | CCTCTGCAGCAGAAGAGG | Promoter cloning |
| proLsRGL1-R | AATTTACGTTAGAGTTACTTCACC | Promoter cloning |
| proLsWRKY70-F | TATCCTTCACCTTGTCGT | Promoter cloning |
| proLsWRKY70-R | GCTTATTTGTCCATTCTT | Promoter cloning |
| q18S-F | GTGAGTGAAGAAGGGCAATG | RT-qPCR analysis |
| q18s-R | CACTTTCAACCCGATTCACC | RT-qPCR analysis |
| qLsRGL1-F | AGTGGAGAAGCAGCAGCAGAGG | RT-qPCR analysis |
| qLsRGL1-R | CGACGAACTTCCGGCGACATC | RT-qPCR analysis |
| qLsWRKY75-F | AAGTGGTGGGTGTTGGTTGTGATAC | RT-qPCR analysis |
| qLsWRKY75-R | CTACTTGGCTCCTTGTTTGGAATGC | RT-qPCR analysis |
| qLsNAC092-F | TTCCGATTCCATCCAACAGACGAAG | RT-qPCR analysis |
| qLsNAC092-R | CTCACCGAGGTTTGCTCTCCATG | RT-qPCR analysis |
| qLsNAC02-F | TGCCGGAATCGAAACTGGAATTGG | RT-qPCR analysis |
| qLsNAC02-R | GTGTCTTCAAACTTGATGGGATGCG | RT-qPCR analysis |
| qLsERF109-F | TTGTTCAAGCAGATCAGCCGAGAAG | RT-qPCR analysis |
| qLsERF109-R | CGTACCTAGCCATAGCCGTTCTTG | RT-qPCR analysis |
| qLsWRKY46-F | ACGATGGTGGAAGGCCCTCTTAG | RT-qPCR analysis |
| qLsWRKY46-R | AACAACCTTGTACGTGTCGATGGG | RT-qPCR analysis |
| qLsWRKY30-F | AAGCCCATGTAGCGAAGATTCAGAC | RT-qPCR analysis |
| qLsWRKY30-R | ACTCCATCTCTCCCCTCACTTTCAC | RT-qPCR analysis |
| qLsWRKY70-F | GGAGGCAACCCGTCTTCATCTTG | RT-qPCR analysis |
| qLsWRKY70-R | CCCAGAATAAGCCCGTGGTGATAC | RT-qPCR analysis |
| qLsERF025-F | GGAAGCGGCGGAGTTGATGAAG | RT-qPCR analysis |
| qLsERF025-R | ATGCGTCTCTCCCTCGTCACC | RT-qPCR analysis |
| qLsGAI-F | CGGCGGCGAGTAACCAAGATAAG | RT-qPCR analysis |
| qLsGAI-R | CACCCGATTCAAACCGAGTCTTCC | RT-qPCR analysis |
| qLsGA20OX2-F | ACCACCACGAAGTCCTCCCATC | RT-qPCR analysis |
| qLsGA20OX2-R | CGGTGATGACATCGGTGTTCTGG | RT-qPCR analysis |
| qLsGASA1-F | CTCCTCCAACTTGTCCAATCCTTCC | RT-qPCR analysis |
| qLsGASA1-R | ACCTTGCTGCACATGACCCTTTAC | RT-qPCR analysis |
| qLsGA3OX1-F | CCGCTTGTCAGTCGCTTATCTCTAC | RT-qPCR analysis |
| qLsGA3OX1-R | TGCCAAGATACTCGCTCCATGTAAC | RT-qPCR analysis |
| qLsPAT1-F | CCAACGCCTCGGAGCTTACATG | RT-qPCR analysis |
| qLsPAT1-R | GTCCCACTCCCAAGGTCATTGC | RT-qPCR analysis |
| qLsGA20OX1-F | CCACTACCACAACTTCGTGTTCCTC | RT-qPCR analysis |
| qLsGA20OX1-R | TTCTCGACAAGCAGCATCAACTAGC | RT-qPCR analysis |
| qLsGID1B-F | AACCACTGAGCACCACCGAAATC | RT-qPCR analysis |
| qLsGID1B -R | GTGAGACGGCGGCAGAATGTG | RT-qPCR analysis |
| qLsABI5-F | CGGTAATGGAGGAGACAGCAATGG | RT-qPCR analysis |
| qLsABI5-R | CTAACCACACCAGCCTTCACCAAG | RT-qPCR analysis |
| qLsABI4-F | TTCTTCTCCTTCCACCTCCTCTTCC | RT-qPCR analysis |
| qLsABI4-R | AAAGTTGGCTCCTCCTCCTACGG | RT-qPCR analysis |
| qLsFUL-F | AGCCAAGAAGACCGAACTCATGTTG | RT-qPCR analysis |
| qLsFUL-R | GCAGGTGACATCTCCATCTCAGC | RT-qPCR analysis |
| qLsFT-F | TCCTGATGCTCCAAGTCCTAGTGAC | RT-qPCR analysis |
| qLsFT-R | TCCCGTGGTCGCTGGTATATCG | RT-qPCR analysis |
| qLsSOC1-F | GGGAAGACTCAAATGCGGAGGATTG | RT-qPCR analysis |
| qLsSOC1-R | CAAAGCAACCTCAGCATCACAAAGC | RT-qPCR analysis |
| qLsLFY-F | CGTCTTCACAAGTGGTTGGAGGAG | RT-qPCR analysis |
| qLsLFY-R | TCAGTCACAATGAACGGATGCTCTC | RT-qPCR analysis |
| qLsFLM-F | AGCGGAGAATGTGTTTGGGATTGG | RT-qPCR analysis |
| qLsLFM-R | AGAGTCAGATGTTGCGGCGAATC | RT-qPCR analysis |
| LsRGL1-F | UUGUACUACACAAAAGUACUG | In situ hybridization(ISH) |
| LsRGL1-R | TGAGCAGCTTGAAATGGTGATGGGTGAAGATGGGATTTTGCAACT | In situ hybridization(ISH) |
| pGADT7-LsRGL1-EcoR1-F | ATGGCCATGGAGGCCAGTGAAATGATACATGCCACCATGGGGACT | Vector construction |
| pGADT7-LsRGL1-BamH1-R | CATCTGCAGCTCGAGCTCGATGGCATCTCCAAGCAGAAGCAGC | Vector construction |
| pHIS2-proLsWRKY70-EcoRI-F | ATACGACTCACTATAGGGCGTATCCTTCACCTTGTCGT | Vector construction |
| pHIS2-proLsWRKY70-BamH1-R | GATTCGCGAACGCGTGAGCTCCGCTTATTTGTCCATTCTT | Vector construction |
| pBI101-proLsWRKY70-BamHI-F | CTGCAGGTCGACTCTAGAGGATATCCTTCACCTTGTCGT | Vector construction |
| pBI101-proLsWRKY70-SmaI-R | CATAAGGGACTGACCACCCGGGCTTATTTGTCCATTCTT | Vector construction |
| pBI121-LsRGL1-EcoRI-F | CATTTGGAGAGAACACGGGGGACTATGATACATGCCACCATGGGGACT | Vector construction |
| pBI121-LsRGL1-SacI-R | TGAACGATCGGGGAAATTCGAGCTGCATCTCCAAGCAGAAGCAGC | Vector construction |
| Biotin-LsWRKY70-a-F | TATCCTTCACCTTGTCGTGGCCATCAC | Vector construction |
| Biotin-LsWRKY70-a-R | TTCCATAATTAAATTCGATACATAAGA | Vector construction |
| Biotin-LsWRKY70-b-F | GTATCGAATTTAATTATGGAAAAACCA | Vector construction |
| Biotin-LsWRKY70-b-R | TGACGTTGTGGATGCCTATATTTTCAA | Vector construction |
| Biotin-LsWRKY70-c-F | ATATAGGCATCCACAACGTCACCGTCT | Vector construction |
| Biotin-LsWRKY70-c-R | AATTACCAAAAATATCAGTAGAGTTAA | Vector construction |
| Biotin-LsWRKY70-d-F | ACTCTACTGATATTTTTGGTAATTAAA | Vector construction |
| Biotin-LsWRKY70-d-R | TGTAGAAAAAGCAAGTGTCCCGAAGGT | Vector construction |
| TRV2-LsWRKY70-EcoR1-F | GATTCTGTGAGTAAGGTTACCGTGAGTCTGACCCAACATCAATCGAT | Vector construction |
| TRV2-LsWRKY70-BmaH1-R | GAGACGCGTGAGCTCGGTACCGCCCAAGCATGCCCATCATCAAT | Vector construction |
| OE-LsRGL1-M13F | TGTAAAACGACGGCCAGT | Transgenic identification |
| OE-LsRGL1-M13R | CAGGAAACAGCTATGACC | Transgenic identification |
| RNAi-LsRGL1-35S promoterF | CTATCCTTCGCAAGACCCTTC | Transgenic identification |
| RNAi-LsRGL1-GUSR | CATAGGCGTCTCGCATATCTC | Transgenic identification |

**Supplementary Table S3 Summary of RNA-Seq data from leaf lettuce libraries**

| **Sample** | **Raw reads** | **Clean reads** | **Clean bases** | **Error rate(%)** | **Q20(%)** | **Q30(%)** | **GC content(%)** | **Total mapped** |
| --- | --- | --- | --- | --- | --- | --- | --- | --- |
| OE_1 | 48671748 | 48446190 | 7.17G | 0.02 | 98.5 | 95.12 | 43.84 | 45803904(94.55%) |
| OE_2 | 47028904 | 46754988 | 6.91G | 0.02 | 98.32 | 94.65 | 43.87 | 43989305(94.08%) |
| OE_3 | 49753984 | 49493586 | 7.34G | 0.02 | 98.42 | 94.88 | 43.75 | 46932251(94.82%) |
| OE_4 | 47514694 | 47274348 | 7.00G | 0.02 | 98.38 | 94.78 | 43.86 | 44155890(93.4%) |
| RNAi_1 | 49814512 | 49549658 | 7.31G | 0.02 | 98.51 | 95.17 | 45.23 | 46978571(94.81%) |
| RNAi_2 | 45305866 | 45058344 | 6.70G | 0.02 | 98.4 | 94.87 | 45.16 | 42636084(94.62%) |
| RNAi_3 | 46279946 | 46010020 | 6.81G | 0.02 | 98.48 | 95.08 | 45.45 | 43646741(94.86%) |
| RNAi_4 | 47498558 | 47268632 | 7.00G | 0.02 | 98.51 | 95.13 | 45.55 | 44862027(94.91%) |
| WT_1 | 49131172 | 48861886 | 7.22G | 0.02 | 98.59 | 95.41 | 45.58 | 45075280(92.25%) |
| WT_2 | 47108660 | 46858162 | 6.93G | 0.02 | 98.4 | 94.85 | 45.45 | 44442715(94.85%) |
| WT_3 | 49651232 | 49375498 | 7.30G | 0.02 | 98.48 | 95.1 | 45.56 | 46949404(95.09%) |
| WT_4 | 50831926 | 50573638 | 7.47G | 0.02 | 98.51 | 95.18 | 45.6 | 47964152(94.84%) |

**Supplementary Table S4 Elements analysis of *LsRGL1* promoter from leaf lactuca**

| **Site Name** | **Matrix sequence** | **Position** | **Strand** | **Motif annotation** |
| --- | --- | --- | --- | --- |
| G-Box | CACGTT | 102 | - | cis-acting regulatory element involved in light responsiveness |
| ABRE | ACGTG | 103 | + | cis-acting element involved in the abscisic acid responsiveness |
| ABRE | ACGTG | 941 | - | cis-acting element involved in the abscisic acid responsiveness |
| MBS | CAACTG | 1544 | + | MYB binding site involved in drought-inducibility |
| GATA-motif | AAGATAAGATT | 858 | - | part of a light responsive element |
| GATA-motif | AAGATAAGATT | 1221 | + | part of a light responsive element |
| GARE-motif | TCTGTTG | 126 | + | gibberellin-responsive element |
| Box III | atCATTTTCACt | 354 | - | protein binding site |
| TGA-element | AACGAC | 879 | + | auxin-responsive element |
| G-box | TACGTG | 941 | - | cis-acting regulatory element involved in light responsiveness |
| G-box | TAACACGTAG | 1797 | + | cis-acting regulatory element involved in light responsiveness |
| TCA-element | TCAGAAGAGG | 526 | + | cis-acting element involved in salicylic acid responsiveness |
| GT1-motif | GGTTAA | 1106 | + | light responsive element |

**Supplementary Table S5 Elements analysis of *LsWRKY70* promoter from leaf lactuca**

| **Site Name** | **Matrix sequence** | **Position** | **Strand** | **Motif annotation** |
| --- | --- | --- | --- | --- |
| TCT-motif | TCTTAC | 531 | + | part of a light responsive element |
| G-box | CACGAC | 13 | - | cis-acting regulatory element involved in light responsiveness |
| G-box | CACGTC | 1713 | - | cis-acting regulatory element involved in light responsiveness |
| CCAAT-box | CAACGG | 275 | + | MYBHv1 binding site |
| I-box | gGATAAGGTG | 212 | - | part of a light responsive element |
| circadian | CAAAGATATC | 1388 | - | cis-acting regulatory element involved in circadian control |
| ABRE | ACGTG | 1714 | + | cis-acting element involved in the abscisic acid responsiveness |
| GT1-motif | GTGTGTGAA | 426 | - | light responsive element |
| ARE | AAACCA | 480 | + | cis-acting regulatory element essential for the anaerobic induction |
| ARE | AAACCA | 1372 | - | cis-acting regulatory element essential for the anaerobic induction |
| CGTCA-motif | CGTCA | 946 | + | cis-acting regulatory element involved in the MeJA-responsiveness |
| CGTCA-motif | CGTCA | 1262 | + | cis-acting regulatory element involved in the MeJA-responsiveness |
| CGTCA-motif | CGTCA | 1304 | + | cis-acting regulatory element involved in the MeJA-responsiveness |
| CGTCA-motif | CGTCA | 1574 | + | cis-acting regulatory element involved in the MeJA-responsiveness |
| CGTCA-motif | CGTCA | 1625 | + | cis-acting regulatory element involved in the MeJA-responsiveness |
| CGTCA-motif | CGTCA | 1649 | + | cis-acting regulatory element involved in the MeJA-responsiveness |
| CGTCA-motif | CGTCA | 1735 | + | cis-acting regulatory element involved in the MeJA-responsiveness |
| ACE | CTAACGTATT | 371 | + | cis-acting element involved in light responsiveness |
| TGACG-motif | TGACG | 946 | - | cis-acting regulatory element involved in the MeJA-responsiveness |
| TGACG-motif | TGACG | 1262 | - | cis-acting regulatory element involved in the MeJA-responsiveness |
| TGACG-motif | TGACG | 1304 | - | cis-acting regulatory element involved in the MeJA-responsiveness |
| TGACG-motif | TGACG | 1574 | - | cis-acting regulatory element involved in the MeJA-responsiveness |
| TGACG-motif | TGACG | 1625 | - | cis-acting regulatory element involved in the MeJA-responsiveness |
| TGACG-motif | TGACG | 1649 | - | cis-acting regulatory element involved in the MeJA-responsiveness |
| TGACG-motif | TGACG | 1735 | - | cis-acting regulatory element involved in the MeJA-responsiveness |
| GTGGC-motif | CAGCGTGTGGC | 1629 | - | part of a light responsive element |
| MRE | AACCTAA | 887 | + | MYB binding site involved in light responsiveness |
