# Supplementary figures and images for: GA signaling protein LsRGL1 interacts with the abscisic acid signaling-related gene LsWRKY70 to affect the bolting of leaf lettuce
Source: Hortic Res. 2023 Apr 19;10(5):uhad054. doi: 10.1093/hr/uhad054 (PMC10199715; doi:10.1093/hr/uhad054)

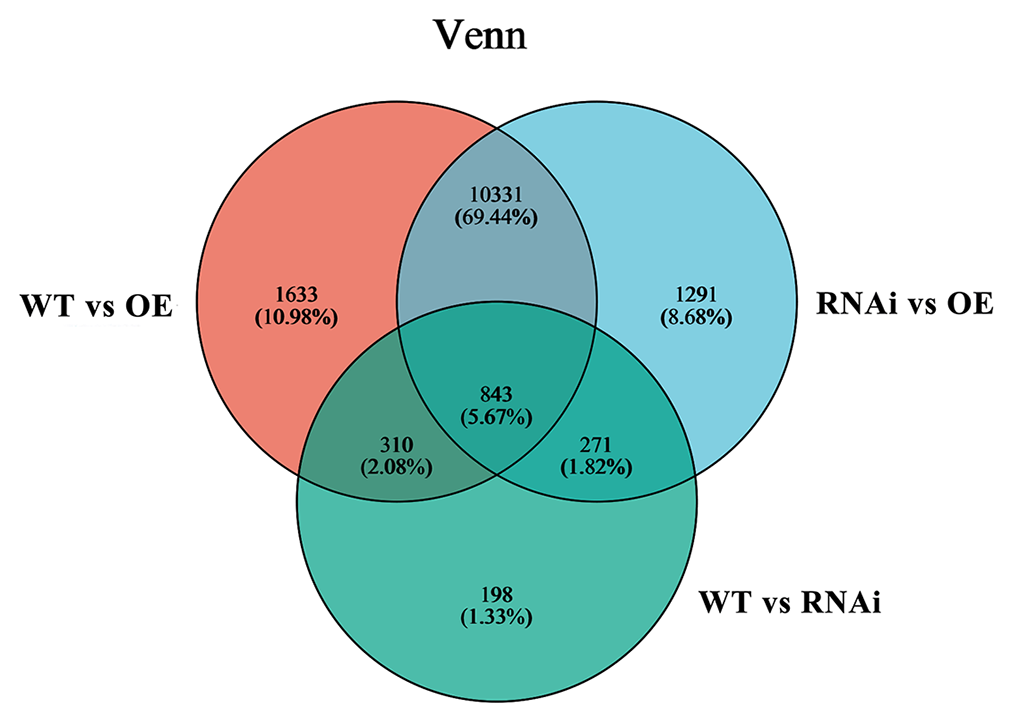

Supplement: Web_Material_uhad054 [file web_material_uhad054.zip › Supplementary Fig2.tif]
